# MiR-367 alleviates inflammatory injury of microglia by promoting M2 polarization via targeting CEBPA

**DOI:** 10.1007/s11626-020-00519-5

**Published:** 2020-11-04

**Authors:** Hui Pei, Qian Peng, Shewei Guo, Yulei Gu, Tongwen Sun, Dong Xu, Yumin Jiang, Jiafeng Xie, Luanluan Zhang, Zhiqiang Zhu

**Affiliations:** 1 Department of Emergency Intensive Care Unit, the First Affiliated Hospital of Zhengzhou University, No.1, East Jianshe Road, Erqi District, Zhengzhou, 450052 Henan Province China; 2 Department of Plastic Surgery, The First Affiliated Hospital of Zhengzhou University, Zhengzhou, China; 3 Department of Neurosurgery, The First Affiliated Hospital of Zhengzhou University, Zhengzhou, China; 4 Department of General Intensive Care Unit, The First Affiliated Hospital of Zhengzhou University, Zhengzhou, China

**Keywords:** miR-367, M2 polarization, Microglia, CEBPA, Intracerebral hemorrhage

## Abstract

MiR-367 was reported to regulate inflammatory response of microglia. CCAAT/enhancer-binding protein α (C/EBPA) could mediate microglia polarization. In this study, we explored the possible roles of miR-367 and CEBPA in intracerebral hemorrhage (ICH). ICH and normal specimens were obtained from the tissue adjacent to and distant from hematoma of ICH patients, respectively. Microglia were isolated and identified by immunofluorescence. The isolated microglia were treated with erythrocyte lysate and randomly divided into 8 groups using different transfection reagents. The transfection efficiency of miR-367 was determined by qRT-PCR. The expressions of M1 and M2 microglia markers were detected by Western blotting. The relationship between CEBPA and miR-367 was confirmed by dual luciferase reporter system. Flow cytometry was performed to determine the level of apoptosis in the cells transfected with miR-367 and CEBPA in erythrocyte lysate–treated microglia. We found that miR-367 expression level was downregulated in ICH specimens. Erythrocyte lysate–treated microglia was successfully established using erythrocyte lysate, as decreased miR-367 expression was observed. Overexpression of miR-367 could significantly decrease the expressions of MHC-ІІ, IL-1β, and Bax, reduced apoptosis rate, and increased the expressions of CD206, Bal-2, and Arg-1 in erythrocyte lysate–treated microglia. CEBPA was proved to be a direct target for miR-367, which could inhibit microglia M2 polarization and increase apoptosis rate. However, in the presence of both CEBPA and miR-367 mimic, the protein and mRNA expressions of CEBPA were decreased, leading to promoted microglia M2 polarization and a decreased apoptosis rate. MiR-367 regulates microglia polarization by targeting CEBPA and is expected to alleviate ICH-induced inflammatory injury.

## Introduction

Hemorrhagic stroke (also known as intracerebral hemorrhage (ICH)) is the most acute and serious cerebrovascular disease, as it has a quick onset and high mortality and disability rates (Shi *et al.* 2016; Zhou *et al.* 2017) and can cause hematoma and secondary pathological processes (Psaila *et al.* 2009; van Asch *et al.* 2010; Tatlisumak *et al.* 2018). ICH causes secondary injury via various pathways, of which inflammatory response is one of the most pivotal pathways (Hamzei Taj *et al.* 2016). Therefore, inhibiting the production of pro-inflammatory mediators is possibly an effective strategy for preventing brain injury after ICH.

Microglia plays an important role in inflammatory response (Shi *et al.* 2016). After ICH, microglia is activated and release inflammatory factors, thereby exacerbating ICH-induced injury (Zhang *et al.* 2017). Increasing evidence showed that microglia with different phenotypes can produce either detrimental or beneficial responses depending on specific environmental signals (Boche *et al.* 2013; Lee *et al.* 2016; Shi *et al.* 2016; Zhang *et al.* 2017). Microglia can be divided into classically activated M1 and alternatively activated M2 according to their surface markers and intracellular cytokines (Boche *et al.* 2013; Lee *et al.* 2016; Shi *et al.* 2016). Classically activated M1 can increase pro-inflammatory cytokines (e.g., IL-1β, TNF-α, iNOS) and M1 marker major histocompatibility complex class ІІ (MHC-ІІ) and aggravate inflammatory response, while alternatively activated M2 can secrete anti-inflammatory cytokines (e.g., Arg-1, IL-4, IL-13) and M2 marker CD206 and exerts an opposite effect to that of M1 microglia (Miron *et al.* 2013; Hamzei Taj *et al.* 2016; Xu *et al.* 2016; Zhang *et al.* 2017). Microglia can change its morphology and express MHC-II, allowing them to function as antigen presenting cells that present neuronal debris as antigen to invade T cells (Yanuck 2019). In agreement with microglia activation, profound morphological changes and MHC-II upregulation occurred upon graft-versus-host disease induction (Mathew *et al.* 2020). M1 transformation was prevented through reducing the release of inflammatory factors of M1 phenotype TNF-α, IL-6, and IL-1β, and increasing the release of cytokines of M2 phenotype, while increasing the expressions of M2 markers (CD206 and Arg-1) in vivo was concomitant with the amelioration of cerebral injury and neurological functions deficits (Han *et al.* 2018). M1 and M2 can alleviate ICH-induced inflammatory response by mediating microglia M2 polarization (Shi *et al.* 2016; Lan *et al.* 2017; Zhou *et al.* 2017). The natural product pinocembrin could reduce the number of M1 microglia without affecting M2 microglia, inhibit neuroinflammation and protect hemorrhagic brain (Lan *et al.* 2017). Shi *et al.* (Shi *et al.* 2016) also found that sinomenine could reduce ICH-induced inflammation by attenuating M1 microglia and promoting M2 microglia. In addition, regulatory T lymphocytes (Tregs) were proved to accelerate brain recovery after ICH through modulating microglia polarization toward M2 phenotype (Zhou *et al.* 2017).

Promoting microglia M2 polarization via targeting control will possibly become a new direction for the treatment of neurological diseases mediated by inflammation. MicroRNAs (miRNAs) are endogenous small RNAs with 18-25 nucleotides in length (Yu *et al.* 2014). Some miRNAs can reduce ICH-induced brain injury by targeting different pathways (Yang *et al.* 2015; Xu *et al.* 2017). For example, inhibiting miR-27b could alleviate ICH-induced brain injury by promoting Nrf2/ARE pathway activation (Xu *et al.* 2017), and miR-233 was proved to reduce inflammatory response via responding to NLRP3 inflammasome after ICH (Yang *et al.* 2015). Recent research showed that CEBPA, which is a transcription factor that mediates the differentiation of pluripotent myeloid progenitor cells into mature granulocytes, could mediate microglia polarization (Yu *et al.* 2017), and that miR-124 attenuated ICH-induced inflammatory injury by increasing M2-polarized microglia through targeting CEBPA (Yu *et al.* 2017). In addition, CEBPA was reported to be a target of miR-367 in the growth regulation of glioma cells (He *et al.* 2018).

Previous study indicated that miR-367 could also reduce the inflammatory response of microglia (Yuan *et al.* 2015). However, whether miR-367 alleviates ICH-induced inflammatory injury by promoting microglia M2 polarization via CEBPA has not been identified. Thus, in this study, we further explored the potential roles of miR-367 and CEBPA in ICH.

## Materials and Methods

### Specimen collection

Thirty ICH patients (16 male and 14 female, aged from 38 to 55 y old) treated by craniotomy in the First Affiliated Hospital of Zhengzhou University between Apr. 2018 and Oct. 2018 were enrolled. The patients were confirmed as having ICH by CT scan or MRI. Patients with traumatic brain injury, secondary brain hemorrhage due to the use of anticoagulant, cerebral vascular malformation hemorrhage, cancer, or other causes, and patients with severe liver and kidney diseases or lung infection were excluded from the study. ICH specimens and normal specimens (each about 1 mm^3^) were obtained from perihematoma and the tissue distant from hematoma during hematoma evacuation, respectively. The collected specimens were stored at 4°C, and examined within 24 h. The study was approved by the Ethics Committee of the First Affiliated Hospital of Zhengzhou University, and informed consent was signed by the participants.

### Microglia isolation and culture

The monocytes THP-1 cells were purchased from the American Type Culture Collection (ATCC, Manassas, VA). Microglia were isolated from the normal specimens and washed with Hank’s balanced salt solution (HBSS, Gibco BRL, Waltham, MA) to remove meninges and visible blood vessels. Then the specimens were minced and incubated with 0.25% trypsin-EDTA solution in phosphate-buffered saline (PBS, Sigma-Aldrich, Billerica, MA) at room temperature for 1 h. The suspension was filtered and centrifuged (at room temperature, 300×*g*, for 5 min) to isolate mixed glia cells. The isolated cells were then plated in a 75-cm^2^ culture flask at 2 × 10^7^ cells per flask in Dulbecco’s modified Eagle’s medium (DMEM, Sigma-Aldrich) containing 10% fetal bovine serum (FBS, Sigma-Aldrich) at 37°C with 5% CO_2_. The medium was changed every 3 d. After 12 d, microglia adhered to the bottom were isolated and cultured in the same way as described above.

### Microglia identification

The cells were washed with 3 ml of PBS and treated with 2 ml of 4% paraformaldehyde at room temperature for 15 min. Then, the cells were washed three times with 4 ml of PBS, and incubated in blocking buffer (5% serum, 0.1% Triton X-100, in PBS) for 30 min. Next, the cells were incubated with 200 μl of anti-CD11b antibody (ab133357, 1:100, Abcam, Cambridge, MA) and diamidino-2-phenylindole (DAPI) in blocking buffer overnight at 4°C and washed three times with 200 μl of blocking buffer. Finally, the cells were incubated with 200 μl of secondary antibody in blocking buffer in the dark for 30 min at room temperature and washed with PBS three times. Microglias were then observed under a fluorescence microscope. Microglia with a purity of higher than 90% were used for the study.

### Preparation of erythrocyte lysate

Healthy blood samples were collected from healthy adults (8 male and 6 female, aged from 40 to 52 yr old) in the First Affiliated Hospital of Zhengzhou University between Apr. 2018 and Oct. 2018. Informed consents for participation in the scientific research were signed by the participants. Single-cell suspensions of erythrocytes were prepared. One milliliter of red blood cell lysing solution was added into 1 × 10^5^ erythrocytes to incubate the cells for 20 min, and then the cells were centrifuged at 2000×*g* for 10 min. After that, the supernatants were used as erythrocyte lysate.

### Cell treatment

Microglia were collected and seeded into 24-well tissue culture plates at a density of 3 × 10^5^ cells/well and then incubated with 10 μl of erythrocyte lysate or PBS for 3 d. Cytokine levels in the supernatants were determined by quantitative real-time PCR (qRT-PCR).

### Transfection

Erythrocyte lysate–treated microglia were randomly divided into 8 groups, namely, MC, M, IC, I, MC + vector, MC + CEBPA, M + vector, and M + CEBPA. Erythrocyte lysate–treated THP-1 monocytes were randomly divided into 4 groups, namely MC, M, IC, and I. Transfection was performed using Lipofectamine^®^ 2000 reagent (Thermo Fisher, Carlsbad, CA) following the manufacturer’s instructions. In brief, 1 × 10^5^ cells were plated in 24-well plates overnight for attachment. The next day, 5 μl of transfection reagent was diluted in 50 μl of Opti-MEM^®^ medium (Thermo Fisher, Carlsbad, CA), while 14 μg of DNA (miR-367 mimic, inhibitor, mimic control, or inhibitor control) (Dharmacon, Inc. Chicago, IL) was diluted in 700 μl of Opti-MEM^®^ medium. Then, 150 μl of diluted DNA was added to 150 μl of diluted Lipofectamine^®^ 2000 reagent and incubated for 5 min at room temperature. Finally, 50 μl of DNA-lipid complex was added to the cells. The sequences of mimic (M), inhibitor (I), mimic control (MC), and inhibitor control (IC) were 3′-UCUCAACGUAUAAUCGUUGUCA-5′, 5′-AGAGUUGCAUAUUAGCAACAGU-5′, 5′-UUUGUACUACACAAAAGUACUG-3′, and 5′-CAGUACUUUUGUGUAGUACAAA-3′, respectively. To determine whether CEBPA was involved in miR-367-mediated microglia polarization, mimic control and vector, mimic control and CEBPA, mimic and vector, or mimic and CEBPA was co-transfected into microglia using Lipofectamine^®^ 2000 reagent. The plasmid vector used for CEBPA transfection was pcDNA3.1. RNA expression level and protein expression level were detected 48 h and 72 h after the transfection.

### Quantitative real-time PCR

Total RNA was isolated using Trizol reagent (Invitrogen, Carlsbad, CA) according to the manufacturer’s instructions. In brief, 100 mg of collected tissues were triturated in liquid nitrogen, added with 1 ml Trizol reagent, and homogenized using a homogenizer. To determine the expressions of erythrocyte lysate–treated cells (2.5 × 10^5^), the growth medium was removed, and 1 ml of Trizol reagent was added to lyse the cells, and the lysate was pipetted to be homogenized. The treated tissues and cells were incubated for 5 min to fully dissociate the nucleoprotein complexes. Then, 0.2 ml of chloroform was added to the cells and incubated for 3 min. The samples were centrifuged for 15 min at 12,000×*g* at 4°C. The mixture was then separated into a lower red phenol-chloroform phase, a middle phase, and a colorless upper aqueous phase. Contents in the colorless upper aqueous phase were transferred to a new tube by angling the tube at 45°, and 0.5 ml of isopropanol was then added to the aqueous phase. After incubating for 10 min, total RNA was isolated by centrifugation at 12,000×*g* at 4°C for 10 min. Reverse transcription was performed using M-MLV Reverse Transcriptase System (Promega, Madison, WI). Quantitative real-time PCR with Light Cycler (Roche Diagnostics, Mannheim, Germany) and SYBR Green I in SYBRRT-PCR Kit (TaKaRa Biotechnology, Dalian, China) were used to detect mRNA expression. GAPDH served as an internal RNA control for Bcl-2, Bax, and CEBPA. Primers were purchased from Bio Asia Corp. (Shanghai, China), and the sequences were as follows: The Bcl-2 forward: 5′-TTCTTTGAGTTCGGTGGGGTC-3′ and reverse: 5′-TGCATATTTGTTTGGGGCAGG-3′; The Bax forward: 5′-TCCACCAAGAAGCTGAGCGAG-3′ and reverse: 5′-GTCCAGCCCATGATGGTTCT-3′; The CEBPA forward: 5′-GCGGGAACGCAACAACATC-3′ and reverse: 5′-GTCACTGGTCAACTCCAGCAC-3′; The GAPHD forward: 5′-CATGGTCTACATGTTCCAGT-3′ and reverse: 5′-GGCTAAGCAGTTGGTGGTGC-3′. MiR-367 was detected using a miRNA RT kit (ABI) and Taq Man Universal PCR Master Mix (ABI) according to the manufacturer’s instructions. U6 served as an internal control for miR-367. Primers were purchased from Bio Asia Corp. (Shanghai, China) and the sequences were as follows: MiR-367 forward: 5′-ACTGTTGCTAATATGCAACTC-3′ and reverse: 5′-GAACATGTCTGCGTATCTC-3′; U6 forward: 5′-AGAGAAGATTAGCATGGCCCCTG-3′ and reverse: 5′-ATCCAGTGCAGGGTCCGAGG-3′. QRT-PCR reactions were performed under the following conditions: at 50°C for 5 min, at 94°C for 30 s, 40 cycles at 94°C for 5 s, and at 60°C for 30 s. Threshold cycle value (CT) was calculated by the ^ΔΔ^CT method, and the data were analyzed using Light Cycler Software 4.0 (Roche Diagnostics). The experiments were carried out in triplicate.

### Western blotting

Total protein was extracted from cultured cells and quantified using Trizol reagent (Invitrogen) following the protocols. Equal amounts of protein from different cells were separated by 10% SDS-PAGE and transferred to a nitrocellulose membrane (Bio-Rad, Hercules, CA). The membrane was blocked with 5% non-fat milk and incubated with antibodies against MHC-ІІ (ab55152, Abcam), IL-1β (#12242, CST), CD206 (ab64693, Abcam), Arg-1 (#9819, CST), GAPDH (ab8245, Abcam), CEBPA (ab40764, Abcam), and β-actin (ab8245, Abcam) at the concentration of 1:1000 at 4°C overnight. The blots were then incubated with horseradish peroxidase–conjugated secondary antibodies in blocking buffer at room temperature for 2 h. The signal was detected using ECL system (Amersham Pharmacia) and quantified by scanning densitometry and computer-assisted image analyzer.

### Flow cytometry assay of apoptosis

Apoptotic cells were quantified by Annexin V-FITC-propidium iodide (PI) double staining using an Annexin V-FITC apoptosis detection kit. The cells were washed twice with PBS and diluted to a density of 1 × 10^6^ cells/ml. Ten microliters of Annexin V-FITC and 10 μl of PI (20 μg/ml) were added into 100 μl of suspensions and incubated for at least 20 min at room temperature in the dark. Four hundred microliters of PBS binding buffer was then added to each tube. The cells were analyzed using FCM analysis (BD Biosciences Clontech) and CellQuest Pro software version 5.1.

### Dual-luciferase reporter gene assay

The cells at 70–80% confluence were co-transfected in 24-well plates. Then, 0.3 μg of reporter gene plasmid, 0.02 μg of internal control vector pGL4.74 [hRluc/TK] vector (Promega, Fitchburg, WI), 1 μl of transfection agent, and 0.2 μg of expression vector orsi-RNA were mixed together. Forty-eight hours after the transfection, dual-luciferase reporter assay was performed according to the manufacturer’s instructions. The cells were lysed using 1× reporter lysis buffer and harvested. Luminescence was detected by Mithras LB 940 (Berthold Technologies, Oak Ridge, TN). The firefly luciferase activity of the reporter gene plasmid was measured as 1 (m1), while the renilla luciferase activity (internal control) of pGL4.74 [hRluc/TK] vector was measured as 2 (m2). The relative luciferase activity was calculated by the ratio of m1/m2.

### Statistical analysis

The data were analyzed using SPSS version 16.0 (SPSS Inc., Chicago, IL) and shown as means ± standard errors of the means. Statistical analysis was performed by one-way analysis of variance (ANOVA), followed by Dunnett’s post hoc test. *p* < 0.05 was considered statistically significant.

## Results

### MiR-367 expression was downregulated in perihematoma of ICH patients

**Figure 1.**
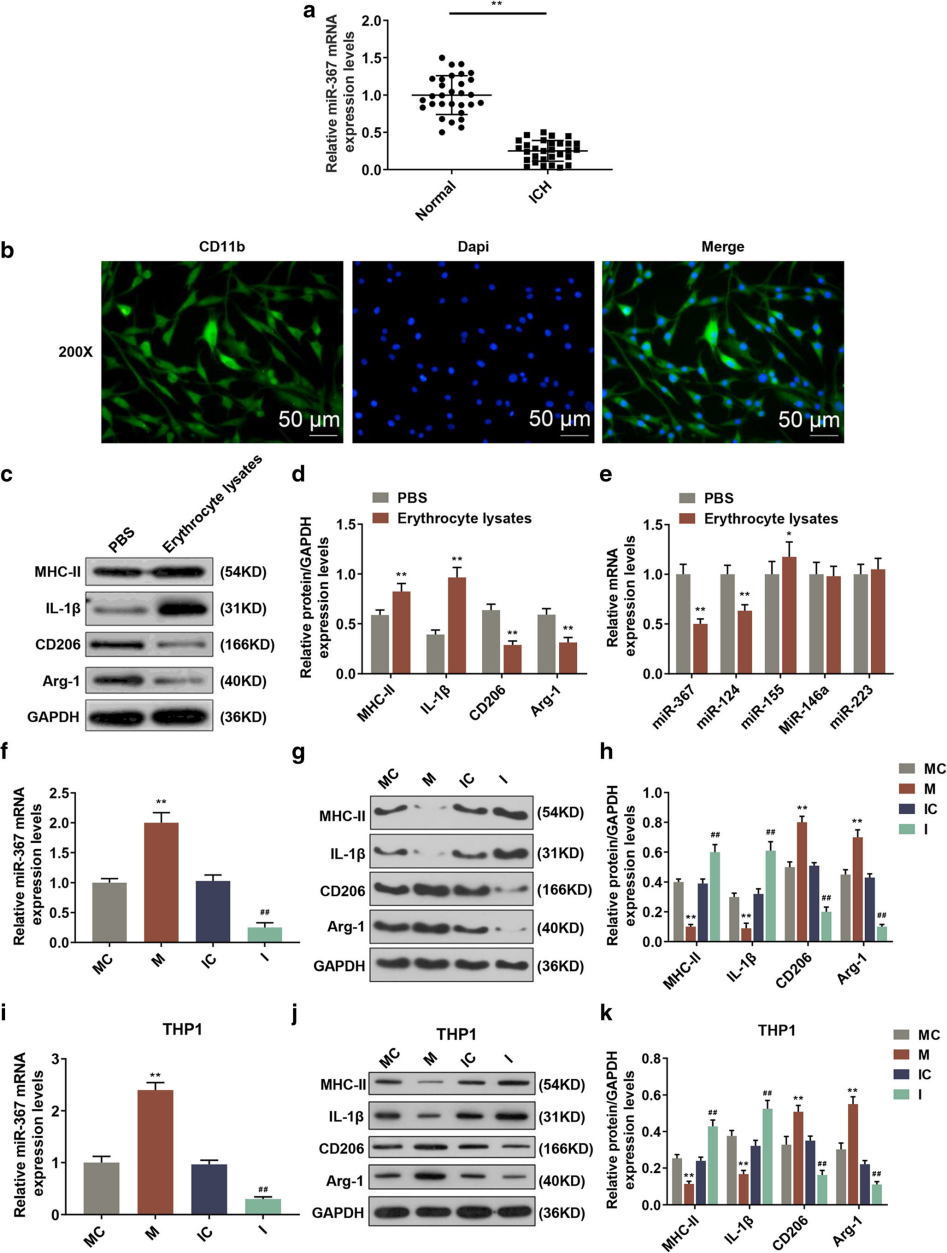
The effects of ICH, erythrocyte lysate, and up/downregulation miR-367 on the mRNA expression level of miR-367. (*a*) The change of miR-367 expression in perihematoma of patients with ICH was determined by qRT-PCR (*vs. normal; **p < 0.01). (*b*) Immunofluorescence of isolated microglia probing for CD11b in gree channel and DAPI in blue channel. (*c*, *d*) The protein levels of M1 microglia markers (MHC-II and IL-1β) and M2 microglia markers (CD206 and Arg-1) were detected by Western blotting assay (*vs. PBS; **p < 0.01). (*e*) QRT-PCR for miRNAs from microglia treated with erythrocyte lysate or PBS for 3 d was applied (*vs. PBS; **p < 0.01). (*f*) QPCR was used to detect the effects of mimic and inhibitor on the mRNA expression level of miR-367 (*vs. MC; ^#^vs. IC; **^/##^*p* < 0.01). (*g*, *h*) The protein levels of M1 microglia markers (MHC-II and IL-1β) and M2 microglia markers (CD206 and Arg-1) were detected by Western blotting assay. (*i*) QPCR was used to detect the effects of mimic and inhibitor on the mRNA expression level of miR-367 in THP-1 cells (*vs. MC; ^#^vs. IC; **^/##^*p* < 0.01). (*j*, *k*) The protein levels of M1 microglia markers (MHC-II and IL-1β) and M2 microglia markers (CD206 and Arg-1) were detected by Western blotting assay in THP-1 cells. Data were shown as mean ± SD from three independent experiments and analyzed by Dunnett’s *t* test. ICH, intracerebral hemorrhage; qRT-PCR, quantitative real-time polymerase chain reaction; DAPI, 4′,6-diamidino-2-phenylindole; PBS, phosphate-buffered solution; MC, mimic control; M, mimic; IC, inhibitor control; I, inhibitor; SD, standard deviation

QRT-PCR was performed to detect the expression level of miR-367 in ICH specimens and normal specimens. As shown in Fig. 1*a*, compared with normal specimens, the level of miR-367 in ICH specimens was reduced significantly (*p*< 0.01).

### Microglia identification

Microglia were isolated to establish the ICH cell culture model. Immunocytochemistry was performed using CD11b as a marker of microglia and DAPI as a marker of cell nucleus to identify isolated microglia. The mergence showed that microglia and cell nuclei could perfectly correspond to each other (Fig. 1B), suggesting that the culture isolated was pure microglia. As some cells were in different division stages with different brightness, so some cells which were brighter than the others, which might be a limitation.

### Erythrocyte lysate–treated microglias were established

Erythrocyte lysate–treated microglias were established (Yu *et al.* 2017) to verify the specific change of miR-367 level. First, we found that erythrocyte lysate significantly increased the protein expressions of M1 microglia markers (MHC-ІІ and IL-1β) and reduced those of M2 microglia markers (CD206 and Arg-1) (Fig. 1*c, d*). Then, the levels of miRNAs were detected by qRT-PCR. Compared with the PBS-treated group, the level of miR-367 in erythrocyte lysate–treated microglia was significantly decreased, the level of miR-124 slightly decreased, and miR-155 slightly increased, while the level of miR-146a and miR-223 changed a little (Fig. 1*e*). The change of miR-367 was the highest among detected miRNAs and was consistent with the results in ICH patients, suggesting that erythrocyte lysate–treated microglia were successfully established.

### MiR-367 mimic promoted microglia M2 polarization and reduced apoptosis in erythrocyte lysate–treated microglia

**Figure 2.**
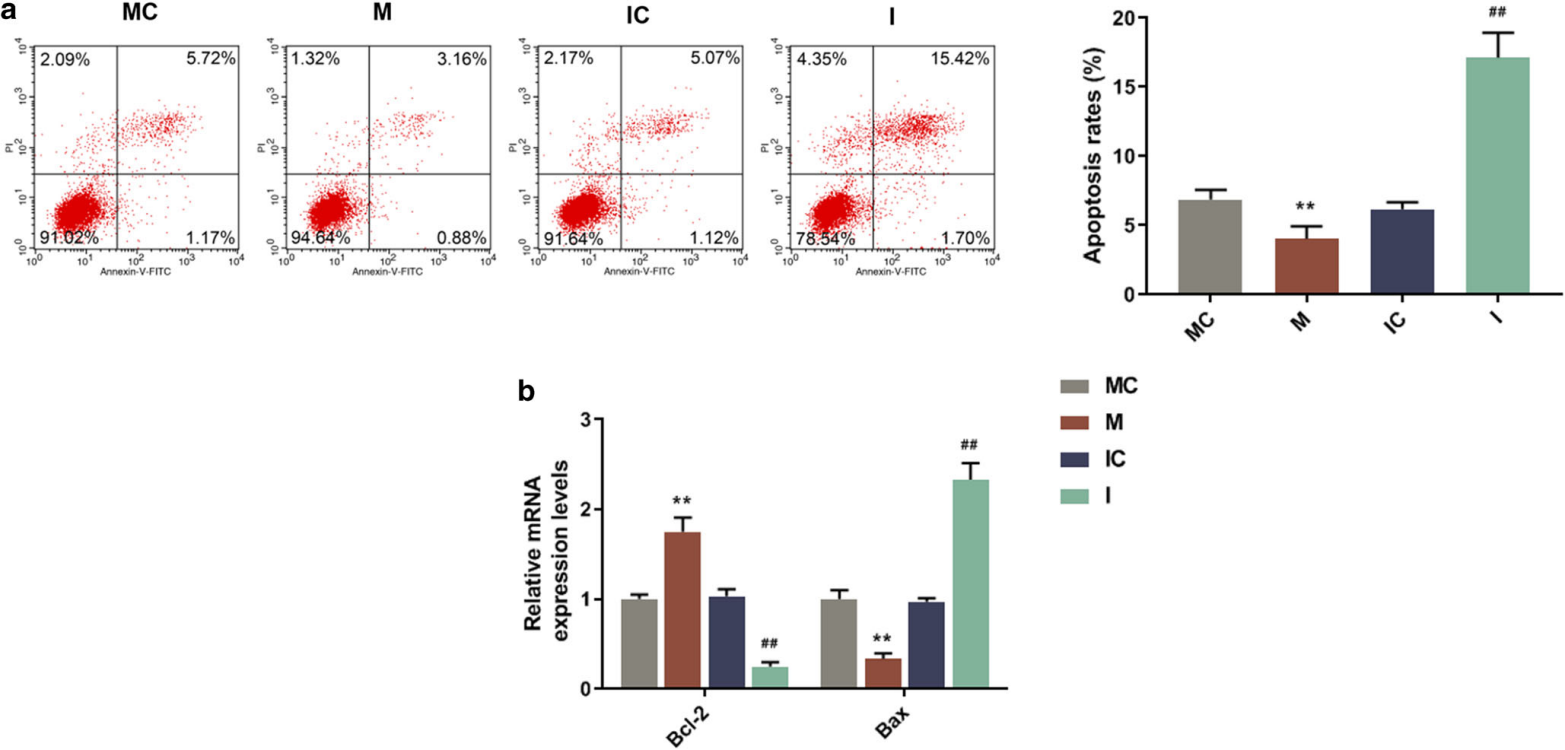
The effects of up/downregulated miR-367 on microglia polarization and cell apoptosis. (*a*) Flow cytometry was used to assess the effect of miR-367 mimic or inhibitor on the apoptosis of erythrocyte lysate-treated microglia. (*b*) QRT-PCR was performed to determine the effect of miR-367 mimic or inhibitor on the mRNA expression levels of apoptosis-related genes. Data were shown as mean ± SD from three independent experiments and analyzed using Dunnett’s *t* test (*vs. MC; ^#^vs. IC; **^/##^*p* < 0.01). MHC-II, major histocompatibility complex-II; IL-1β, interleukin-1β; CD206, mannose receptor; Arg-1, arginase-1; Bcl-2, B cell lymohoma-2

To further explore the function of miR-367 in microglia polarization and apoptosis in erythrocyte lysate treated microglia, mRNA level, protein expression and cell apoptosis were determined by qRT-PCR, Western blotting and flow cytometry, respectively. MiR-367 mimic and inhibitor were used to upregulate and downregulate miR-367 in erythrocyte lysate–treated microglia (Fig. 1*f*), respectively. We found that miR-367 mimic significantly reduced the protein expressions of M1 microglia markers (MHC-ІІ and IL-1β) and increased those of M2 microglia markers (CD206 and Arg-1) (Fig. 2*g, h*), indicating that overexpressed miR-367 promoted microglia M2 polarization. Other than microglia, miR-367 mimic and inhibitor were used to upregulate and downregulate miR-367 in erythrocyte lysate–treated THP-1 monocytes (Fig. 1*i*), respectively. We found that miR-367 mimic significantly reduced the protein expressions of M1 microglia markers (MHC-ІІ and IL-1β) and increased those of M2 microglia markers (CD206 and Arg-1) in erythrocyte lysate–treated THP-1 monocytes (Fig. 2*j, k*), indicating that overexpressed miR-367 promoted M2 polarization in THP-1 monocytes. Hence, the results indicated that overexpressed miR-367 promoted microglia M2 polarization.

Moreover, miR-367 mimic could also upregulate the level of Bcl-2, downregulate the level of Bax, and reduce apoptosis rate (Fig. 2*a, b*), while miR-367 inhibitor exerted an opposite effect to that of miR-367 mimic in erythrocyte lysate-treated microglia.

### CEBPA was a direct target for miR-367 in microglia

**Figure 3.**
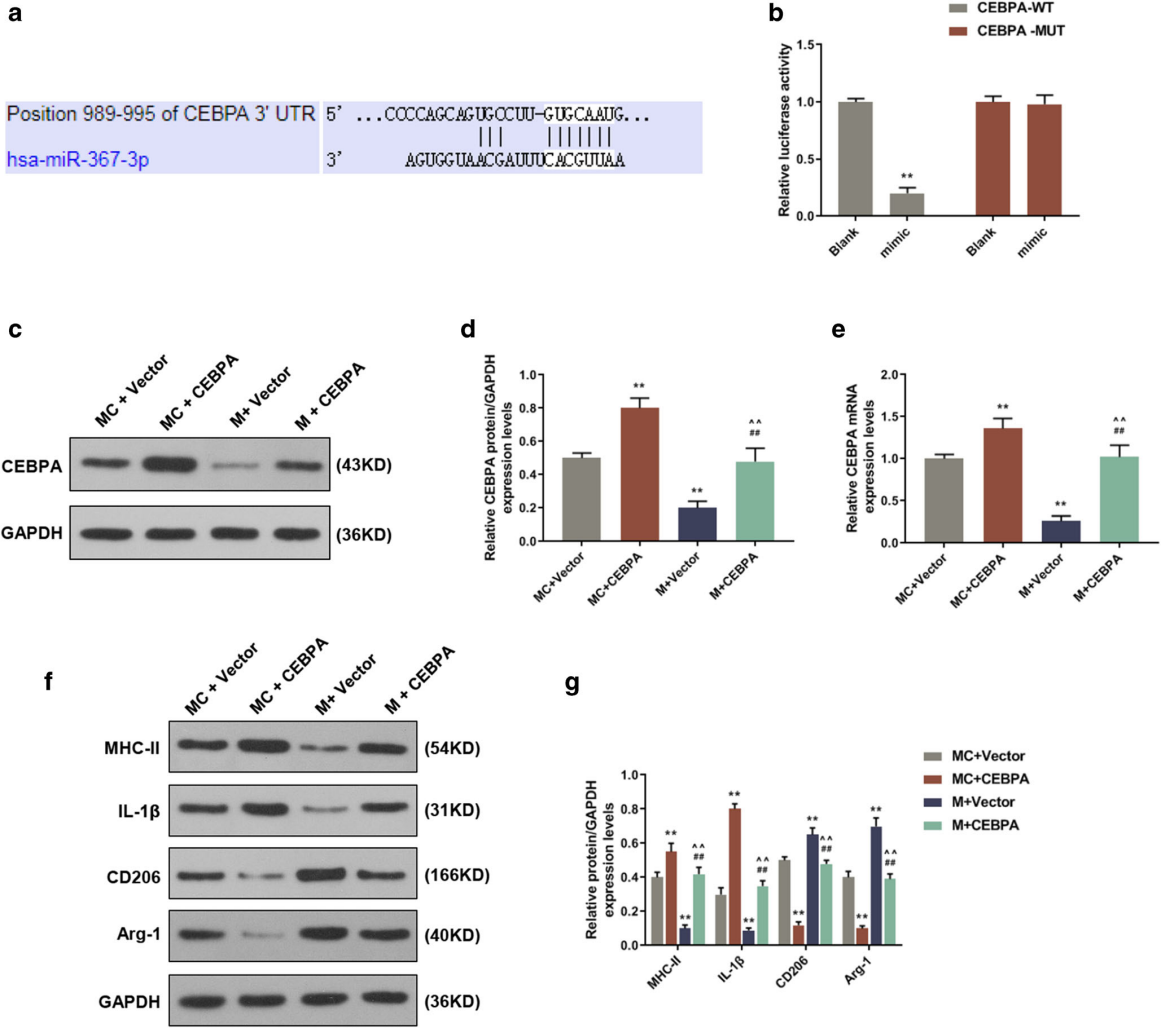
CEBPA was a direct target for miR-367, and miR-367 promoted microglia M2 polarization via CEBPA. (*a*) Sequences of hsa-miR-367-3p recognition region predicted by Targetscan7.2 in the 3′-UTR of CEBPA. (*b*) Luciferase activities of reporter plasmids with wild type or mutated CEBPA 3′-UTR in microglia were examined by transfecting mimics or none (*vs. blank; **p < 0.01). (*c*, *d*) Detection of CEBPA protein levels in microglia by Western blotting (*vs. MC + vector; ^#^vs. M + vector; **^/##^*p* < 0.01). (*e*) QRT-PCR was used to determine the effects of CEBPA and mimic on the mRNA expression level of CEBPA (*vs. MC + vector; ^#^vs. M + vector; **^/##^*p* < 0.01). (*f*, *g*) The protein levels of M1 microglia markers (MHC-II and IL-1β) and M2 microglia markers (CD206 and Arg-1) detected by Western blotting assay (*vs. MC + vector; ^#^vs. M + vector; ^vs. MC + CEBPA; **^/##/^^^*p* < 0.01). Data were shown as mean ± SD from three independent experiments and analyzed by Dunnett’s *t* test.

Target Scan 7.2 (Fig. 3*a*) predicted that CEBPA is a potential target for miR-367, and the direct relationship between CEBPA and miR-367 was further confirmed by dual luciferase reporter system. We found that co-transfecting with miR-367 mimic and wild-type CEBPA 3′-UTR greatly suppressed the luciferase expression level. However, co-transfecting with miR-367 mimic and mutated CEBPA 3′-UTR did not affect the luciferase expression level (Fig. 3*b*). These results demonstrated that CEBPA could be suppressed by miR-367.

### MiR-367 mimic downregulated CEBPA expression in erythrocyte lysate–treated microglia

To explore the effect of miR-367 mimic on the expression of CEBPA in erythrocyte lysate-treated microglia, Western blotting and qRT-PCR assays were performed to detect the protein and mRNA expression levels of CEBPA. We found that transduction of miR-367 mimic downregulated the mRNA and protein expression levels of CEBPA (Fig. 3*c–e*).

### MiR-367 mimic promoted microglia M2 polarization and reduced apoptosis through CEBPA in erythrocyte lysate-treated microglia

**Figure 4.**
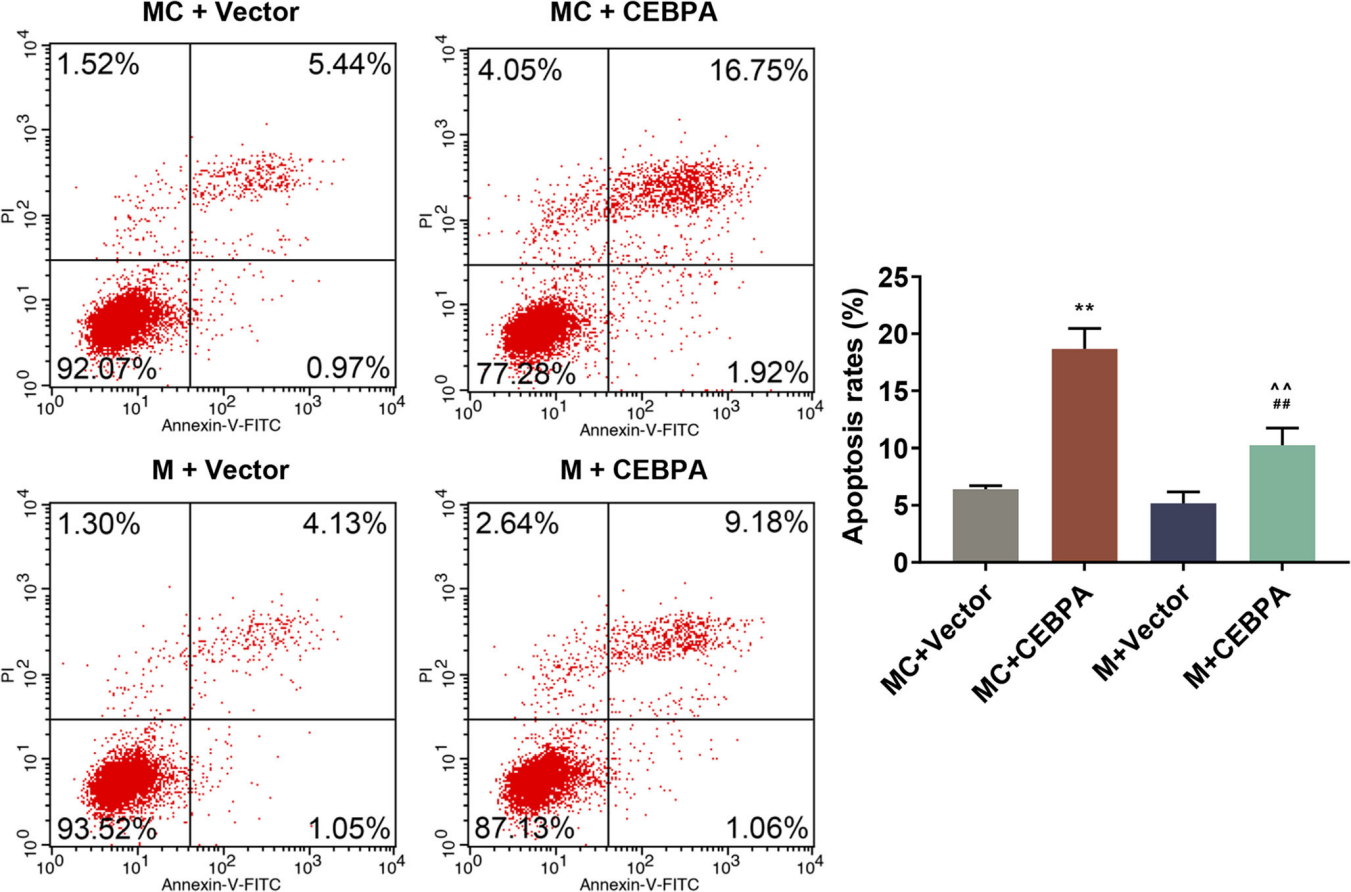
The effects of CEBPA and mimic on microglia apoptosis assessed by flow cytometry. Data were shown as mean ± SD from three independent experiments and analyzed using Dunnett’s *t* test (*vs. MC + vector; ^#^vs. M + vector; ^vs. MC + CEBPA; *p < 0.05; **^/##/^^^*p* < 0.01).

To determine the effects of CEBPA on microglia treated with miR-367 mimic, Western blotting and flow cytometry assays were performed to determine protein expression and cell apoptosis. Obviously, over-expression of miR-367 significantly improved M2 polarization in erythrocyte lysate–treated microglia, while co-transduction with CEBPA produced no effect (Fig. 3*f, g*). Additionally, CEBPA inhibited the reduction of apoptosis rate caused by miR-367 (Fig. 4). These data demonstrated that over-expression of miR-367 promoted microglia M2 polarization and reduced apoptosis via downregulating CEBPA.

## Discussion

ICH is one of the most acute and serious types of stroke. However, there is a lack of a specific treatment for ICH (Hamzei Taj *et al.* 2016). Intensive blood pressure reduction and hemostatic therapy could possibly attenuate ICH-induced brain injury (Wartenberg and Mayer 2015; Morotti *et al.* 2017). Evacuation of hematoma by surgical treatment is another approach to alleviate ICH injury (Mendelow 2015; Rennert *et al.* 2015). Moreover, as secondary injury also contributes to brain injury after ICH (Lee *et al.* 2016), reducing secondary injury is equally important in treating ICH. Studies showed that the process of nerve cell injury after ICH is affected by secondary ischemia in peripheral brain tissue, thrombin release, hemoglobin toxicity, inflammatory response, and apoptosis, and among the above factors, inflammatory response is one of the most important affecting factors (Zhang *et al.* 2017). Numerous reports indicated that the reduction of inflammatory injury had a positive effect on the prognosis of ICH (Lee *et al.* 2016; Yu *et al.* 2017; Zhang *et al.* 2017).

Microglia are inflammatory cells (Shi *et al.* 2016), and specifically, M1 microglia increase inflammation via producing pro-inflammatory cytokines (e.g., MHC-ІІ, IL-1β), while M2 microglia reduce inflammation by releasing neuro-protective factors (e.g., CD206, Arg-1) (Lee *et al.* 2016; Yu *et al.* 2017) (Yuan *et al.* 2015). Therefore, mediating microglia M2 polarization could be explored to treat ICH patients. Studies showed that miRNA expression level changed with microglia phenotypes, and that miRNAs also regulated the polarizaion of microglia (Yu *et al.* 2014; Yang *et al.* 2015; Xu *et al.* 2017).

MicroRNAs (miRNAs) are endogenous small RNAs with 18-25 nucleotides in length (Yu *et al.* 2014), and they can regulate microglia polarization via various pathways. MiR-155 is an M1-related miRNA, and it was reported that miR-155 could affect the interleukin 13–dependent regulation of several genes (SOCS1, DC-SIGN, CCL18, CD23, and SERPINE), thereby affecting the establishment of an M2 phenotype in macrophages (Martinez-Nunez *et al.* 2011). MiR-146 acts as an anti-inflammatory miRNA to target TLR4 signaling and thereby suppresses iNOs and promotes M2 polarization (Vergadi *et al.* 2014). MicroRNA-223 regulates inflammation and brain injury via feedback to NLRP3 inflammasome after intracerebral hemorrhage (Yang *et al.* 2015). MiR-124 was shown to ameliorate ICH-induced inflammatory injury via promoting microglia M2 polarization through CEBPA (Yu *et al.* 2017).

CEBPA has been proved as a tumor-inhibiting factor and is related to various types of cancers (Lourenco and Coffer 2017; Voutila *et al.* 2017). Upregulation of CEBPA might reduce key pro-inflammatory cytokines, thus showing an anti-inflammatory potential (Freire and Conneely 2018; Zhou *et al.* 2019). It was also reported that CEBPA mediated microglia polarization and could be beneficial in reducing ICH-induced inflammatory injury [13]. Recent research indicated that miR-367 could downregulate the inflammatory response of microglia (Yuan *et al.* 2015). Similarly, it was found that miR-367 expression was decreased in perihematoma of patients with ICH in our study. MiR-367 belonged to miR-92a family and it was reported barely detectable in macrophages (Lai *et al.* 2013). To investigate the mechanisms of miR-367 and CEBPA in ICH, cell culture of erythrocyte lysate–treated microglia was successfully established by erythrocyte lysate treatment, which was confirmed by the increase of M1 microglia markers (MHC-ІІ and IL-1β) and decrease of M2 microglia markers (CD206 and Arg-1). The representative miRNAs (miR-367, miR-124, miR-155, miR-146a, miR-223) being related to M1/M2 was detected, and miR-367 level was found to significantly decrease by erythrocyte lysate treatment. Then, we found that upregulating miR-367 could significantly decrease the protein expressions of M1 microglia markers (MHC-ІІ and IL-1β) and increase those of M2 microglia markers (CD206 and Arg-1) in erythrocyte lysate–treated microglias and monocytes. Cell apoptosis was a common mechanism in many bio-activities (Zhang *et al.* 2019). Moreover, we found that miR-367 mimic could also upregulate Bcl-2, downregulate Bax, and reduce apoptosis rate. However, miR-367 inhibitor exerted an opposite effect to that of miR-367 mimic. CEBPA was predicted as a direct target for miR-367 in microglia, and it could promote microglia M1 polarization (MHC-ІІ and IL-1β), inhibit microglia M2 polarization (CD206 and Arg-1), and increase apoptosis rate. The effect of CEBPA on inflammatory factors in our study was opposite to the observations before (Freire and Conneely 2018), which might depend on different mechanisms. However, when CEBPA and miR-367 co-existed, the protein and mRNA expressions of CEBPA were decreased, resulting in a reduction of microglia M1 polarization, an increase of microglia M2 polarization, and a lower apoptosis rate. MHC-ІІ, IL-1β, CD206, and Arg-1 studied in our research are representative factors that reflect microglia M1 and M2 polarization types. More specific high molecular markers for microglia M1 and M2 polarization types could be studied in the future. In addition, more in vivo experiments are needed in future study to further verify the results. Besides, we did not study humoral molecules from erythrocytes or the molecular interaction between humoral molecules and microglia nor did we apply monocytes or macrophages to compare with the effect of miR-367 on microglia or even compare miR-367 with other miRNAs. These works would be conducted in the future.

In conclusion, our findings demonstrated that CEBPA aggravated inflammatory injury caused by erythrocyte lysate, while miR-367 could attenuate the injury by promoting microglia M2 polarization via targeting downregulated CEBPA. The results of our study provide a new feasible strategy for alleviating secondary injury in ICH.
